# Antimicrobial effects of microwave‐induced plasma torch (MiniMIP) treatment on *Candida albicans* biofilms

**DOI:** 10.1111/1751-7915.13459

**Published:** 2019-07-01

**Authors:** Oliver Handorf, Uta Schnabel, André Bösel, Thomas Weihe, Sander Bekeschus, Alexander Christian Graf, Katharina Riedel, Jörg Ehlbeck

**Affiliations:** ^1^ Leibniz Institute for Plasma Science and Technology (INP) Felix‐Hausdorff‐Str. 2 17489 Greifswald Germany; ^2^ School of Food Science and Environmental Health, College of Sciences and Health Technological University Dublin Cathal Brugha Street D01 HV58 Dublin Ireland; ^3^ Institute of Microbial Physiology and Molecular Biology University of Greifswald Felix‐Hausdorff‐Str. 8 17489 Greifswald Germany

## Abstract

The susceptibility of *Candida albicans* biofilms to a non‐thermal plasma treatment has been investigated in terms of growth, survival and cell viability by a series of *in vitro* experiments. For different time periods, the *C. albicans* strain SC5314 was treated with a microwave‐induced plasma torch (MiniMIP). The MiniMIP treatment had a strong effect (reduction factor (RF) = 2.97 after 50 s treatment) at a distance of 3 cm between the nozzle and the superior regions of the biofilms. In addition, a viability reduction of 77% after a 20 s plasma treatment and a metabolism reduction of 90% after a 40 s plasma treatment time were observed for *C. albicans*. After such a treatment, the biofilms revealed an altered morphology of their cells by atomic force microscopy (AFM). Additionally, fluorescence microscopy and confocal laser scanning microscopy (CLSM) analyses of plasma‐treated biofilms showed that an inactivation of cells mainly appeared on the bottom side of the biofilms. Thus, the plasma inactivation of the overgrown surface reveals a new possibility to combat biofilms.

## Introduction

Non‐thermal plasmas (NTPs) are also known as non‐equilibrium plasmas, honouring the fact that the cooling of ions and uncharged particles is more effective than an energy transfer of energetically excited electrons to the latter particles. Thus, the gaseous environment is not heated up, which is contrary to thermal plasmas (Fridman, Friedman *et al.*, 2008). Today, NTPs play an increasingly important role in both medicine and industry. They combine the advantage of non‐thermal operation and high antimicrobial activity (Surowsky, Schlüter *et al.*, 2015). Hence, NTPs are used in today’s medicine for many different applications such as wound healing (Shekhter, Serezhenkov *et al.*, 2005; Ghaffari, Jalili *et al.*, 2007), cell detachment as well as reattachment (Kieft, Darios *et al.*, 2005; Kieft, Kurdi *et al.*, 2006) and biological decontamination (Laroussi, Alexeff *et al.*, 2000; Laroussi, Mendis *et al.*, 2003). Due to their capability of biological decontamination, NTPs have gained interest in many industries. In the dairy and food industry, biofouling caused by microbial biofilms is a serious problem, which leads to a considerable loss of resources. This is caused not only by the microbial contamination of end‐products such as meat, fruits and vegetables (Kumar and Anand, 1998), but also by increased corrosion rates at processing surfaces and increased fluid frictional resistance and heat flow across the surface (Criado, Suarez *et al.*, 1994).

The predominant microbial life cycle implies attachment to solid surfaces and the formation of three‐dimensional, multicellular aggregates called biofilms, which are embedded in a self‐produced, extracellular matrix (ECM; Steenackers, Parijs *et al.*, 2016, Serra, Hidalgo‐Bastida *et al.*, 2017). Especially, food‐borne pathogens and spoilage microorganisms like *Candida albicans* prefer biofilm formation on stainless steel, aluminium, glass, polytetrafluoroethylene (PTFE) seals and polyamide (PA) material, which are typically found in food‐processing environments (Herald and Zottola, 1988; Mafu, Roy *et al.*, 1990; Notermanns, Dormans *et al.*, 1991). *Candida* spp., for example, can often be isolated from conveyor tracks of the food and beverage industry (Loureiro and Malfeito‐Ferreira, 2003; Brugnoni, Lozano *et al.*, 2007).

Economic losses in the food and healthcare sector as well as a more sensitive public awareness put food safety into the spotlight. Consequently, European countries implemented the Good Manufacturing Practice (GMP) and Hazard Analysis Critical Control Point (HACCP) for food industries. As a consequence, in 2016 the European Food Safety Authority was able to identify 49,950 food‐borne outbreaks resulting in illness, 3869 hospitalizations and 20 deaths (European Centre for Disease Prevention and Control & European Food Safety Authority, 2017). Notably, 99,392 outbreaks resulting in illness were reported in the United States, leading to 2625 hospitalizations and 115 deaths (Centers for Disease Control and Prevention, 2016). Conventional methods like the mechanical removal of biofilms by high‐pressure cleaners or brushing and wiping indeed lead to a loss of biomass, but not to an efficient extent. Due to intense and brief‐acting shear forces, the majority of the biomass is removed but a thin biofilm most likely remains which further grows much denser with an increased resistance (Liu and Tay, 2002). Additionally, in older mature biofilms, significant components of the biofilm often remain, causing the biofilm to grow again (Jang, Rusconi *et al.*, 2017). The treatment of biofilms with biocides is just as problematic as the biofilms show increased resistance to these biocides compared to their planktonic counterparts. This is mainly due to the extracellular matrix which prevents deep penetration of the biofilm with the biocides (Bridier, Dubois‐Brissonnet *et al.*, 2011). This leads to low concentrations in the deeper layers of the biofilm, which in turn leads to increased horizontal gene transfer of resistance genes (Jutkina, Marathe *et al.*, 2018). Thus, there is a strong need for novel strategies combating biofilms in the food industry. These range from the treatment of biofilms with special oils (Kerekes, Vidács *et al.*, 2015) and enzymes (Meireles, Borges *et al.*, 2016) to bacteriophages. The treatment of microorganisms with NTPs is a constantly growing field in which new insights into the effects are constantly being gained (Sladek, Filoche *et al.*, 2007; Koban, Holtfreter *et al.*, 2011; Xu, Tu *et al.*, 2011; Alkawareek, Algwari *et al.*, 2012; Ermolaeva, Sysolyatina *et al.*, 2015; Flynn, Higginbotham *et al.*, 2015). Results obtained in the here presented study suggest that the microwave‐induced plasma torch (MiniMIP) is a powerful tool for microbial decontamination. In order to meet the industrial requirements, it is of particular importance to find standardized parameters for the plasma source. Despite antimicrobial effects of a different plasma source on *C. albicans* having been shown (Handorf, Weihe *et al.*, 2018), no studies exist, which investigated the effects of the MiniMIP on *C. albicans* biofilms up to this date.

## Results

The path of the development from a new plasma source concept to its effective use in a specific application also includes the investigation of a potential antimicrobial impact on surface‐bound biofilms and their basic physical adaptations to it. Consequently, the presented work summarizes test series to determine the antimicrobial effect of the MiniMIP plasma source on eukaryotic biofilms. Due to its ubiquitous presence in medical and food sectors, *C. albicans* has been chosen as a model microbe, because its biofilm formation has been intensely studied and it is contaminant of medical as well as industrial importance (Kabir, Hussain *et al.*, 2012; Morata and Loira, 2017). In particular, *C. albicans* strain SC5314 is known for its rapid vertical growth and could be compared with *Saccharomyces cerevisiae*, a yeast of great importance in the food and beverage industry because of its high sugar consumption and the fermentation of juices into alcoholic end‐products (Battey, Duffy *et al.*, 2002; Walker and Stewart, 2016; Lorenzini, Simonato *et al.*, 2019). This study investigated the viability of the cells [revealed via the fluorescence assay (2.5)] and cellular metabolism [XTT assay (2.6)] and was complemented by the determination of the post‐treatment viability (CFU 2.4). Additionally, we used fluorescence microscopy (2.7), CLSM (2.8) and AFM (2.9) to obtain optical evidence of the plasma influence on the cells. Finally, OES (2.10) was used to give a general overview of the chemical composition of the plasma gas.

### Effects of plasma treatment on the proliferation of the cells

**Figure 1:**
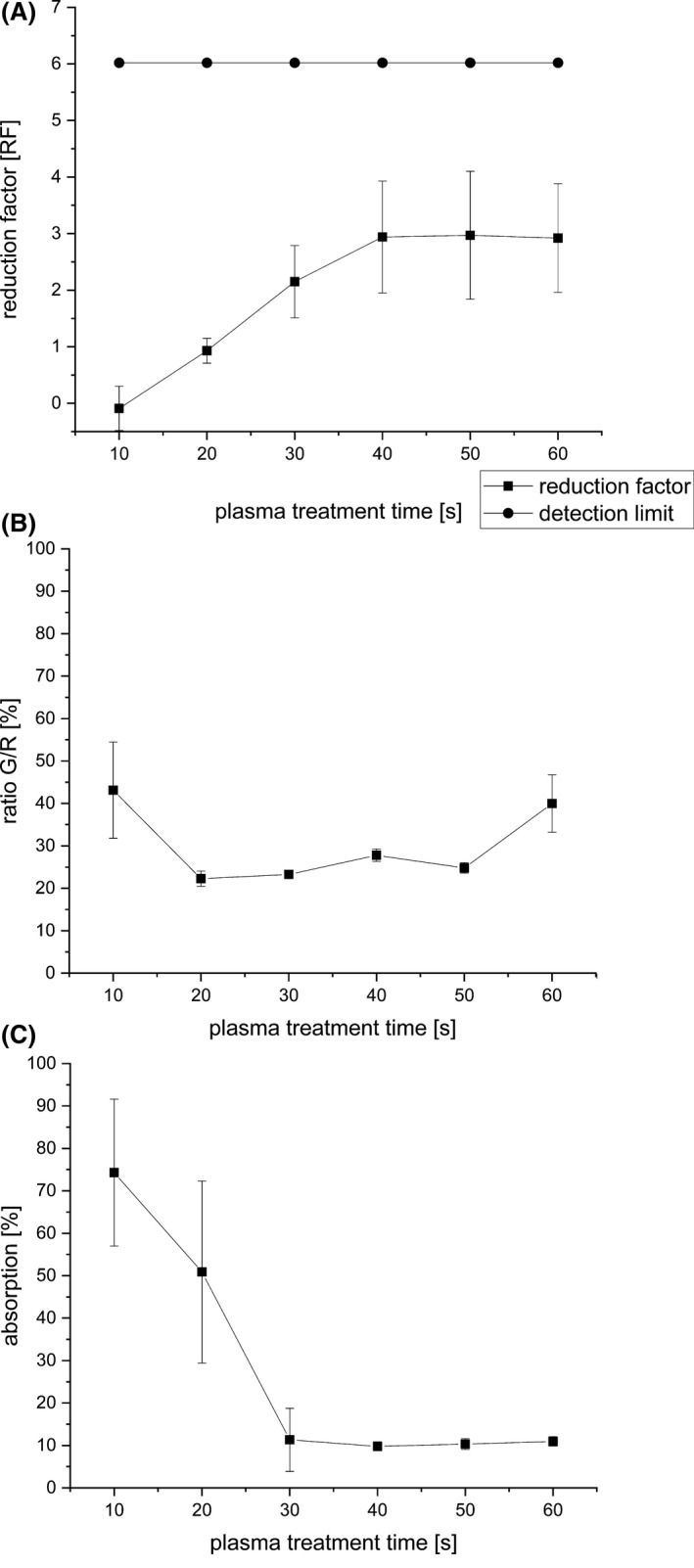
CFU, fluorescence and XTT assay of *C. albicans* biofilms after treatment with the MiniMIP. (A) CFU measurements of the MiniMIP‐treated *C. albicans* biofilms. The line with ● represents the detection limit. The line with ▪ shows the reduction factor (RF) of the different plasma treatment times. The error bars were calculated using the propagation of error and the weighted error. The RF for the 10 s treatment with the MiniMIP shown to intersect the x‐axis was not statistically significant. (B) Fluorescence LIVE/DEAD assay of the MiniMIP‐treated *C. albicans* biofilms. The ratio G/R is defined as the division of the emission of green fluorescence by the emission of red fluorescence. (C) XTT measurements of the MiniMIP‐treated *C. albicans* biofilms. The data points of all measurements represent the weighted mean value of the total population of the treatment time from the quadruple repetition *n* = 6.

A reduction factor (RF) was calculated as the difference between the log_10_ (CFU) of an untreated control and the log_10_ found for the samples after the treatment. The controls hosted in average 10^6^ cells. The RFs quantify the inhibitory effect of the plasma treatment. The treatment with the MiniMIP revealed a RF of 2.97 after a 50 s treatment (Fig. 1A). The RF for the 10 s treatment with the MiniMIP shown to intersect the x‐axis was not statistically significant. A continuous increase in RF could be detected up to a 40 s plasma treatment. Longer plasma treatment times did not lead to a significant increase in RF.

### Effects of plasma treatment on the viability of the cells

The fluorescence LIVE/DEAD assay, which was used to detect the viability of the cells after the plasma treatment, showed a declined G/R ratio from 2.6 to 0.58 after a 20 s MiniMIP plasma treatment (Fig. 1B), which corresponds to a 77% reduction of the G/R ratio. The maximum reduction observed for a MiniMIP treatment was reached after 20 s. Longer treatment times revealed no further changes in the viability.

### Effect of plasma treatment on the metabolism of the cells

The XTT assay, which was used to determine the metabolic activity of the cells after plasma treatment, showed a reduction in the absorption from 2.09 to 0.20 after a 40 s MiniMIP plasma treatment (Fig. 1C). It represented a reduction of 90%. No further decrease in the cell metabolism could be measured for longer treatment times.

### Fluorescence microscopic confirmation of plasma treatment effects

**Figure 2:**
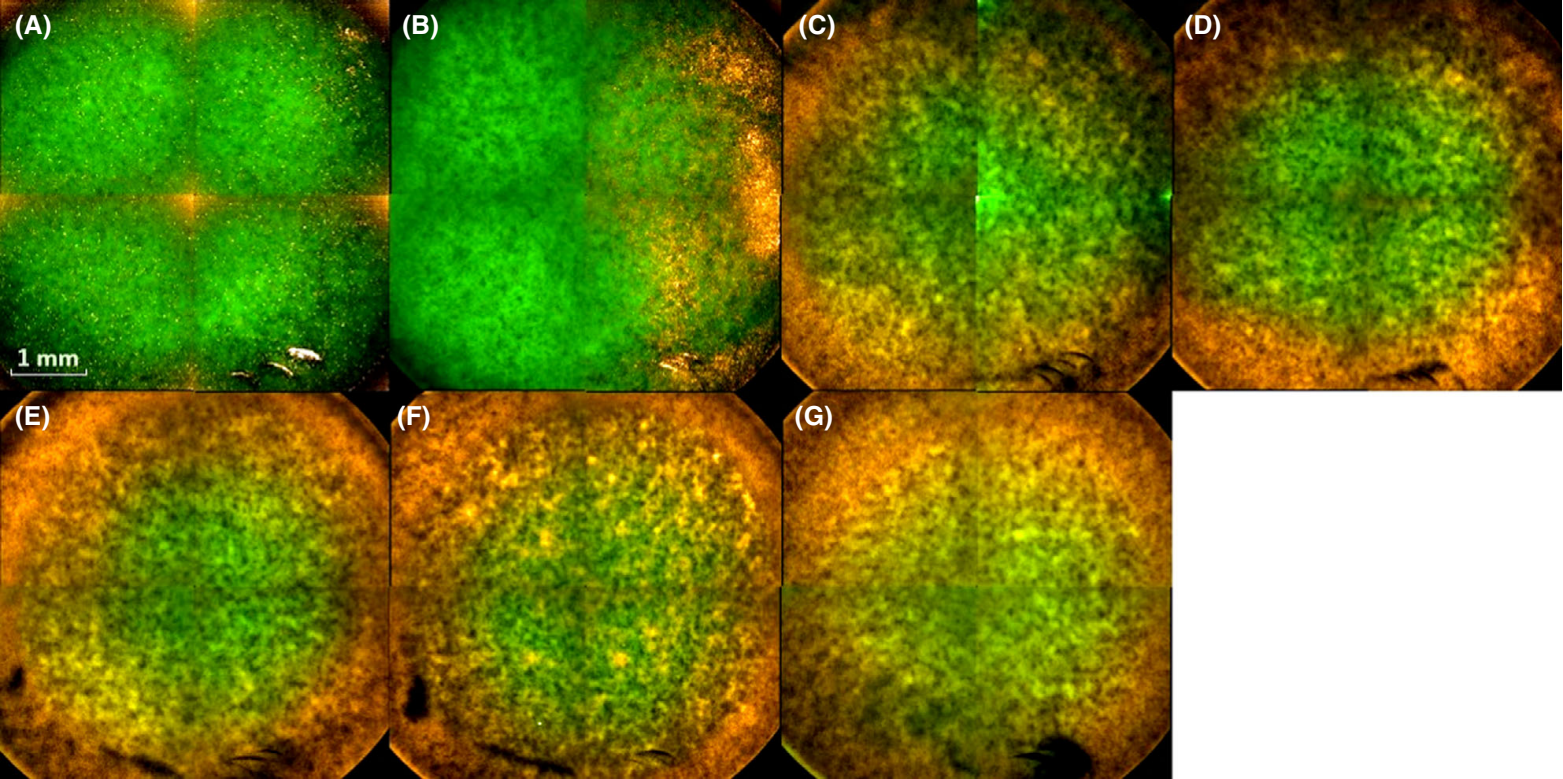
Overview with the Operetta CLS fluorescence microscope of MiniMIP‐treated biofilms. (A) Control; (B) 10 s plasma treatment time; (C) 20 s plasma treatment time; (D) 30 s plasma treatment time; (E) 40 s plasma treatment time; (F) 50 s plasma treatment time; (G) 60 s plasma treatment time. The biofilms were stained with SYTO 9 (green fluorescence for all cells) and propidium iodide (red fluorescence of dead cells). The pictures show inverse images. The scale bar indicates 1 mm.

Fluorescence microscopy indicated a massive impact of the plasma treatment on cells which were located on the bottom of the biofilms already after a 20–30 s treatment (Fig. 2C,D). The biofilm degenerated from its exterior, and the inactivation of cells amplified into the centre of the biofilm with increasing treatment times (Fig. 2E–G). After 60 s treatment time, almost the entire biofilm was affected by the treatment (Fig. 2G).

### CLSM confirmed cell inactivation processes predominantly on the bottom of the biofilms

**Figure 3:**
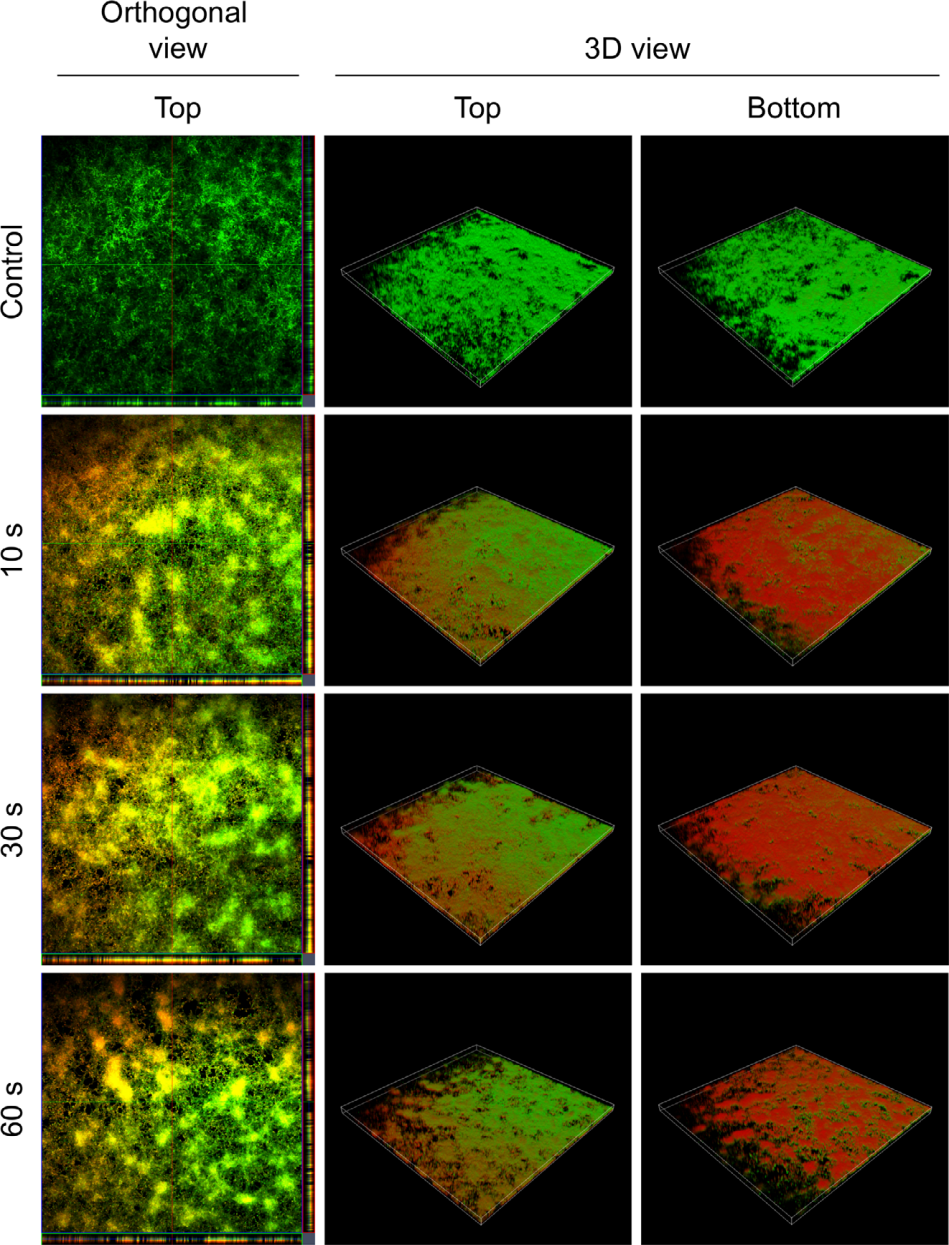
Confocal laser scanning microscopy (CLSM) images of LIVE/DEAD‐stained *C. albicans* biofilms after plasma treatment with the MiniMIP. Left panels show an orthogonal view of the top biofilm layer (horizontal optical sections in the centre and vertical optical sections in the flanking pictures). Central and right panels show 3D images with a top and a bottom view of the biofilms. For each biofilm, an area of 1272.2 µm × 1272.2 µm was visualized.

The influence of a plasma treatment on the three‐dimensional structure of a *C. albicans* biofilm was studied by CLSM (Fig. 3). The right side of the figure shows an inferior view of the 3D biofilm structure, which reveals highly influenced regions already after a 10 s treatment. Although the propagation of inactivated cells in the biofilm was more pronounced in the inferior regions, an inactivation was already noticeable in more peripheral layers of the biofilm, which became obvious in a 3D‐ model (Fig. 3) as well as in orthogonal views of the top layers. With increasing plasma treatment time, almost the entire bottom of the biofilm was inactivated and clear effects were visible on the top layers of the biofilms. Furthermore, increasing separations and holes in the biofilm could be detected with longer treatment times.

### AFM confirmed alterations in the cell morphology of the biofilm after plasma treatment

**Figure 4:**
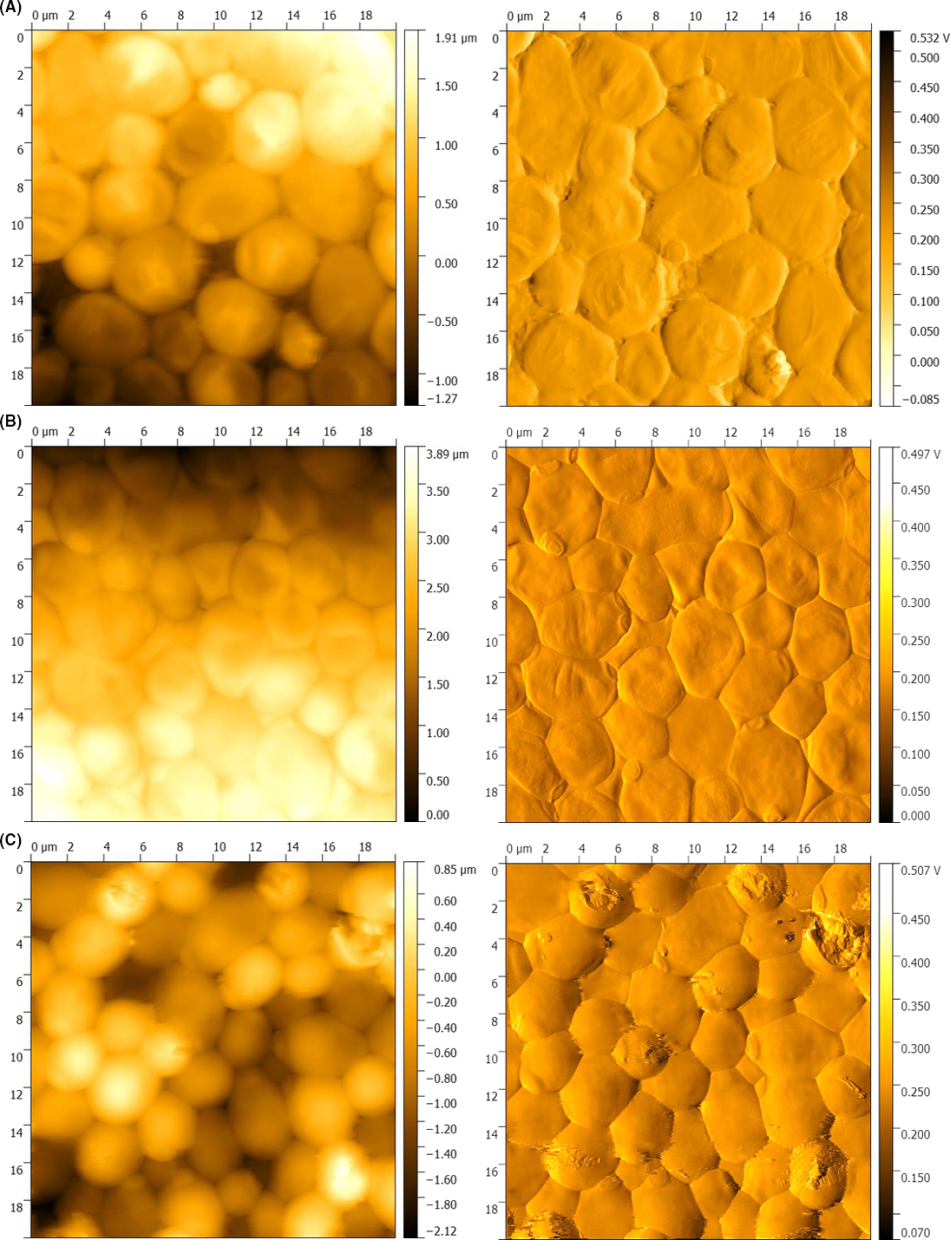
Atomic force microscopy (AFM) images of the MiniMIP‐treated biofilms and untreated controls. The left side shows the topographic image and the right side the error‐signal image of the same spot. (A) Untreated control; (B) 30 s plasma treatment time; (C) 60 s plasma treatment time. The images were acquired in contact mode with a cantilever spring constant of *k* = 0.1–0.6 N/m^2^ and a frequency of 0.4 Hz, the set point at 8 N/m^2^ and an area of 20 µm^2^.

With the aid of AFM, more profound insights in the cell morphological alteration of the biofilm after plasma treatment have been obtained. Due to the method of cantilever visualization, AFM can only visualize the morphological changes of the cells on the surface of the biofilm. The control cells appeared vital and commonly shaped (Fig. 4A). In contrast, cells treated with plasma for 60 s appeared more spherical and partially ruptured with distinct sites fractures. In addition, they were smaller than the control cells (Fig. 4C).

### OES demonstrated the chemical composition of the plasma gas

**Figure 5:**
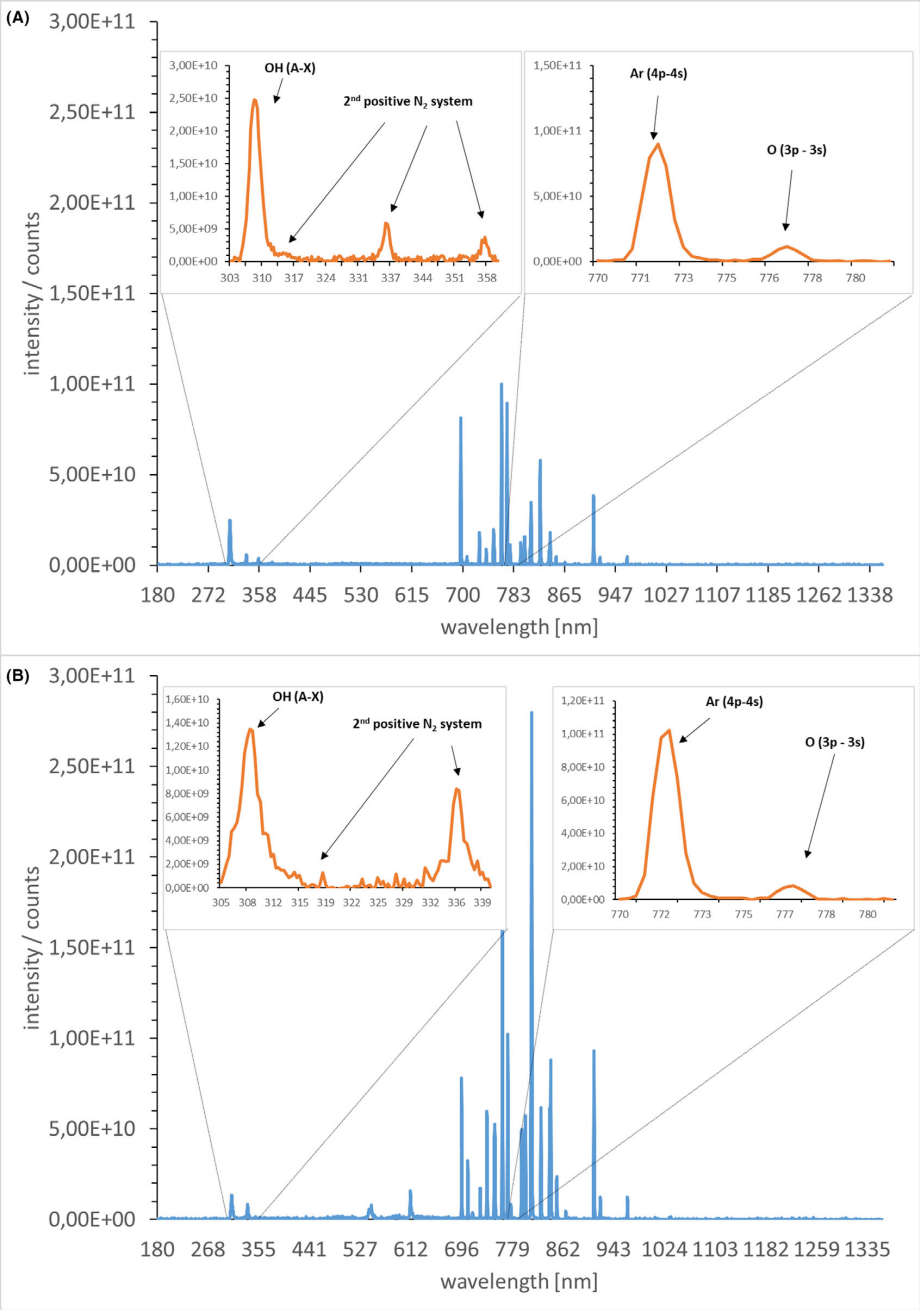
Optical emission spectroscopy (OES) of the radiofrequency plasma jet kINPen09 and the microwave‐induced plasma torch MiniMIP. (A) Emission spectra of the radiofrequency plasma jet kINPen09. (B) Emission spectra of the MiniMIP. The inset boxes represent the respective wavelength region at higher resolution. The emission spectra represent the respective molecules. The measurement results were obtained with LabVIEW and evaluated with MATLAB.

OES detects molecular and atomic emission bands of electromagnetic radiation, which provide information about the plasma composition. Unfortunately, differences in the peak height do not simultaneously indicate differences in the quantity of the molecules. Nevertheless, qualitative statements could be made based on the molecule spectra. Molecular absorption bands for nitrogen and hydroxyl groups were obtained, as well as spectral lines of atomic oxygen and argon in the measurements of the plasma gas. However, additional bands in the range of 520–620 nm were visible in the effluent of the MiniMIP compared to the kINPen09, a well‐studied plasma device with regard to its plasma gas composition (Fig. 5).

## Discussion

Currently, the road in NTP research and development points to an increasing importance for application‐oriented plasma sources because of their differences in design, performance and application, depending on the respective task. The number of different NTP sources including plasma needles (Stoffels, Flikweert *et al.*, 2002; Bora, Aguilera *et al.*, 2018; Mohammed and Abas, 2018), plasma jets (Fricke, Koban *et al.*, 2012; Xu, Shen *et al.*, 2015; Xu, Shen *et al.*, 2017), dielectric barrier discharge (DBD; Pietsch, 2001, de Souza, Neto *et al.*, 2016, Offerhaus, Lackmann *et al.*, 2017) or microwave‐induced plasmas (Jovicevic, Ivkovic *et al.*, 2000; Baeva, Bösel *et al.*, 2012) is constantly growing.

Each of these plasma sources have specific application areas for which they are suited best. Plasma needles, for example, have already been used in dentistry for root canal treatments (Sladek, Stoffels *et al.*, 2004; Goree, Liu *et al.*, 2006). Radiofrequency plasma jet (RFPJ) like the kINPen09 or the new version kINPen MED has already been used for chronic wound healing in medicine (Lademann, Ulrich *et al.*, 2013; Bekeschus, Schmidt *et al.*, 2016). DBD is a promising tool for the microbial decontamination of water (Baroch and Saito, 2011) and has already been applied for the treatment of surfaces (Oehmigen, Hähnel *et al.*, 2010; Baroch and Saito, 2011; Banaschik, Lukeš *et al.*, 2015), for example textiles (Müller, Zahn *et al.*, 2010; Simor, Creyghton *et al.*, 2010). In comparison, the decontamination of biofilms with cold atmospheric pressure plasmas is an emerging field of research with a series of promising results (Machala, Chladekova *et al.*, 2010; Ehlbeck, Schnabel *et al.*, 2011; Misra, Tiwari *et al.*, 2011; Scholtz, Pazlarova *et al.*, 2015; Liguori, Cochis *et al.*, 2017).

So far, microwave‐induced plasmas (MIPs) are mainly used in spectroscopy for the analysis of gas components (Broekaert and Engel, 2006), surface modifications (Jia, Kuraseko *et al.*, 2008) or the processing of biogas (Tippayawong, Chaiya *et al.*, 2015). Not much is known about microwave plasmas which are already used in medical technology apart from SteriPlas (Adtec, Hounslow, UK). The latter technology is based on the microwave plasma technology of the Max Planck Institute for extraterrestrial Physics in Germany. During an 8 year period, chronic wounds of 379 patients were treated with this plasma source and significant reductions in the bacterial count were detected (Isbary, Morfill *et al.*, 2010). Based on the RFs obtained in our experiments, the MiniMIP shown in this study appeared as a versatile tool to combat microbial biofilms in the food and beverage industry.

In general, a distinction has to be made between the type of treatment and the type of plasma generation/ignition (Niemira, 2012). In this work, we used a microwave‐driven plasma source (type of plasma generation) and an indirect treatment of the biofilms (type of treatment). For a comparison, the most reasonable way is to work with plasma sources with a comparable power output and the same working gas. If two plasma sources have to be compared in their antimicrobial effects, they should be used in a standardized assay (Mann, Schnabel *et al.*, 2015; Sarangapani, Patange *et al.*, 2018). For instance, Ehlbeck *et al. *(2008) showed the treatment of contaminated PET bottles with microwave‐induced plasma. In this case, the type of treatment and the type of plasma generation were the same and they reached reduction factors of up to 7. Contrary, comparison of the reduction factors is difficult, as the plasma used in that work had a much higher power and was operated with compressed air instead of argon gas.

The trend for microwave plasmas in microbial decontamination tends more in the direction of plasma‐processed air (PPA) or plasma‐treated water (PTW) which subsequently affect the microorganisms (Schnabel, Andrasch *et al.*, 2014; Thirumdas, Kothakota *et al.*, 2018; Schnabel, Handorf *et al.*, 2019). Especially in terms of industrial manufacturing, PPA and PTW offer advantages over a conventional plasma treatment. For instance, not all areas which need to be treated are easily accessible for plasma devices. In this context, washing and flushing processes with PTW or PPA can offer a decisive advantage.

Indirect treatment of microorganisms where the effluent has been brought into a short distance over the biofilms is published for plasma jets or DBDs (Maisch, Shimizu *et al.*, 2012; Khan, Lee *et al.*, 2016; Handorf, Weihe *et al.*, 2018). If only the achieved reduction factors are considered for the same treatment time regardless of the type of plasma generation and type of application, RFs of 0.6 for the kINPen09, 2.9 for the hollow electrode dielectric discharge (HDBD) and 2.3 for the volume dielectric discharge (VDBD) were obtained after 1 min treatment time (Koban, Matthes *et al.*, 2010). In relation to these results, the MiniMIP showed the strongest inactivation with a reduction factor of 2.92 after 1 min treatment time (Fig. 1A).

In the present study, three different methods (CFU, fluorescence and XTT assay) were used to investigate the viability of *C. albicans* cells organized in a biofilm, after a plasma treatment. However, these methods do not reveal the same effects on the cells. The CFU shows the ability of the cells to proliferate after treatment. The fluorescence assay indicates membrane damage of the cells, and the XTT assay monitors the metabolic activity of the cells after treatment. The results of the methods cannot be directly compared. Not only based on the current study, the increasing importance of viable but non‐culturable (VBNC) cells becomes more and more obvious but should be discussed more nuanced, and it is essential that the results of several different methods are considered together. Concretely, together they give a comprehensive overview which makes it possible to exclude a VBNC status as far as possible (Ramamurthy, Ghosh *et al.*, 2014; Saprykina, Bolgova *et al.*, 2016; Bolgova, Saprykina *et al.*, 2017). The results (Fig. 1) showed that already 20 s after treatment the cells had significant membrane damages and their metabolic activity was strongly reduced. Their ability to proliferate was also severely restricted.

**Figure 6:**
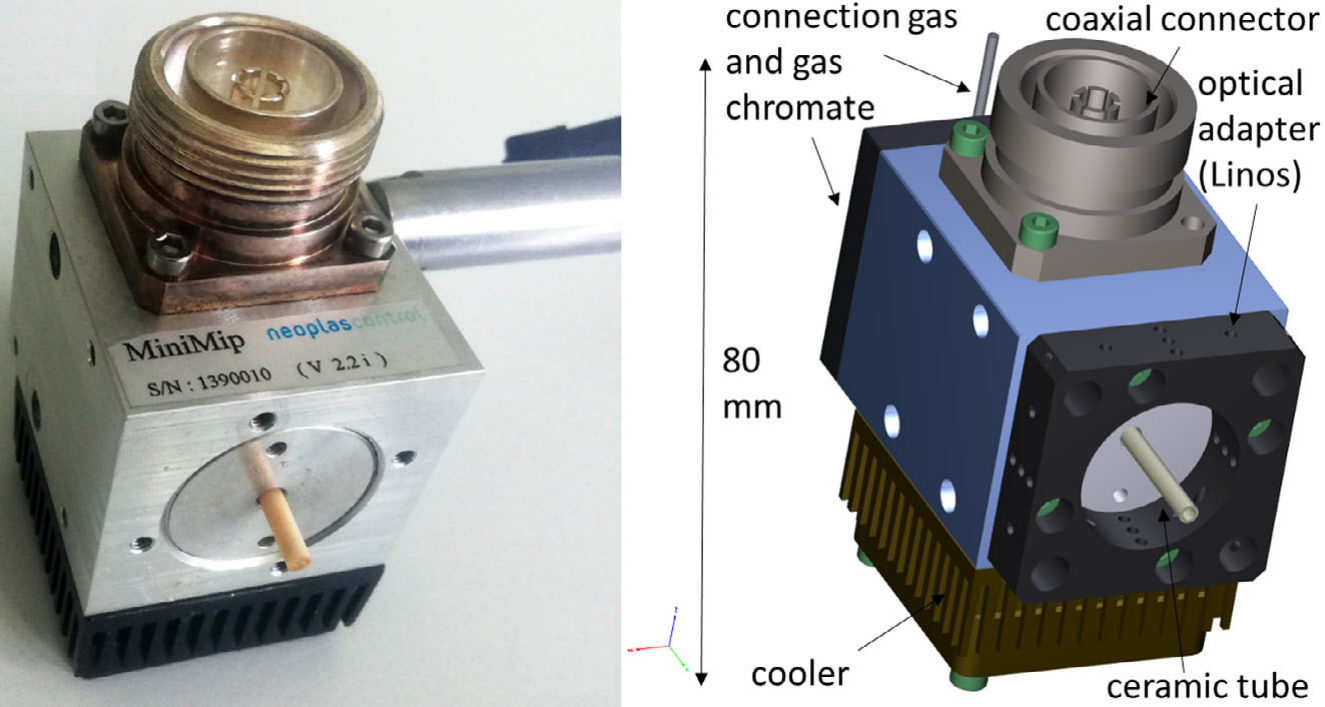
Structure of the microwave‐driven plasma torch MiniMIP. The ceramic tube leads completely into the inside of the housing, where it is encased by an aluminium tube. The ignition of the plasma takes place at the front edge of the aluminium tube, and plasma propagation is driven by the surrounding microwave field and the gas flow along the longitudinal axis of the ceramic tube leading to a small plasma plume of about 10 mm in length and 3 mm in diameter outside the device. Left: microwave‐driven plasma torch MiniMIP; right: schematic structure of the MiniMIP.

In terms of three‐dimensional effects, RFPJ treatments showed centralized spots of dead cells while the majority of biofilm cells were not affected by plasma treatment (Handorf, Weihe *et al.*, 2018). This occurred mainly due to the relatively fine and centralized effluent of plasma jets. In contrast, a very broad effect has been demonstrated in the treatment of biofilms with the MiniMIP, which rather spreads from the marginal areas to the centre and affected the complete biofilm already after 20–40 s (Fig. 2C–E). Most publications showed that the biofilm structures with increasing treatment times were either inactivated on their surfaces or completely inactivated during the course of the treatment (Pei, Lu *et al.*, 2012; Traba and Liang, 2015; Delben, Zago *et al.*, 2016). This could have been a result of the higher power of the plasma sources or the longer treatment times.

It is very likely that the applied plasma treatment times of 10–60 s mirror the dynamic range. In the indirect treatments, the formed reactive oxygen species (ROS) and reactive nitrogen species (RNS) played a major role in inactivation (Klampfl, Isbary *et al.*, 2012; Xu, Shen *et al.*, 2015; Ziuzina, Boehm *et al.*, 2015). There may have been an increase in the concentration of ROS/RNS in the liquid residues of the biofilm that have accumulated on the bottom of the biofilm. In combination with water, RNS such as nitrates and nitrites are to be expected when treating with the MiniMIP. Consequently, PTW was generated at the bottom layers of the biofilm and predominantly led to an inactivation in that area. This was also indicated in treatments with the kINPen09 in the same experimental set‐up (Handorf, Weihe *et al.*, 2018).

The AFM images revealed differences in the cell morphology between a treatment with the MiniMIP or the kINPen09 (Handorf, Weihe *et al.*, 2018). It is most likely caused by the device‐dependent reaction pathways, which lead to ROS/RNS (Yusupov, Neyts *et al.*, 2012; Gilmore, Flynn *et al.*, 2018). However, more in‐depth investigations of the MiniMIP plasma gas constituents have not yet been completed. The PLexc microwave plasma comes closest to the gas physics of the MiniMIP (Pipa, Andrasch *et al.*, 2012). Investigations of the gas physics of this plasma source have shown that RNS is mainly produced during the latter process (Schnabel, Handorf *et al.*, 2019). This knowledge was additionally supported by the temperature‐dependent dissociation rates of the different gas molecules (Drost, 1980). Investigations have already shown that the temperature in the plasma discharge area within the MiniMIP is above 2000°C, where ROS were already dissociated and mainly RNS still exist (Baeva, Bösel *et al.*, 2012). Therefore, the OES spectra in this paper serve as an overview of the gas molecules.

Notably, plasma treatment could overcome various limitations of conventional antimycotic drugs. For instance, it is well‐known that reactive species of plasma gases are able to overcome the barrier of the ECM and inhibit cells in the biofilm or even completely degrade the ECM (Delben, Zago *et al.*, 2016; Modic, McLeod *et al.*, 2017; Gilmore, Flynn *et al.*, 2018). *C. albicans* exploits a range of resistance mechanisms to conventional antimicrobial treatment strategies. Its resistance is primarily due to the activity of efflux pumps, the production of an ECM and the presence of recalcitrant persister cells during biofilm growth (Nobile and Johnson, 2015). The efflux pumps of *C. albicans* comprise two major classes: the ATP‐binding cassette transporter superfamily and the major facilitator class (Anderson, 2005; Cowen, 2008). The ECM represents a mechanical barrier to drugs and thus leads to higher drug resistance of *C. albicans* cells embedded in biofilms (Baillie and Douglas, 2000; Al‐Fattani and Douglas, 2006). Persister cells are a small subset of metabolically dormant yeast cells in biofilms that are extremely resistant to antimycotics (LaFleur, Kumamoto *et al.*, 2006). Comparative studies between plasma treatment of *C. albicans* biofilms and treatment with antimycotics or chemical disinfectants showed a stronger reduction in the CFU during plasma treatment and indicated no correlation between plasma effects and efflux pumps of the pathogen (Koban, Matthes *et al.*, 2010). Furthermore, it could be shown that pre‐treatment with plasma even significantly increases the effect of antimycotics on the pathogens (Sun, Yu *et al.*, 2012).

The results of our work offer new promising fields of application for MIPs. The results of the MiniMIP compared to already well‐investigated plasma sources showed a stronger reduction of *C. albicans* biofilms in a shorter treatment time despite a larger distance of the plasma source to the surface of the biofilm (Handorf, Weihe *et al.*, 2018). The MiniMIP is therefore a promising new tool in the field of plasma‐based antimicrobial decontamination of biofilms. Although the investigation of the effect of MiniMIP on prokaryotic and eukaryotic biofilms is still in its infancy, the results shown here are highly motivating and represent a solid basis for further investigations and applications of this innovative plasma source.

## Conclusion

Our results show the influence of plasma treatment of *C. albicans* biofilms with the microwave‐induced plasma source MiniMIP for the first time. A stronger influence of the MiniMIP on the biofilms could be shown within a shorter plasma treatment time and greater distance compared to already commercially available and well‐studied plasma sources, for example the RFPJ kINPen09. The composition of the plasma gases can be of decisive importance for the different results shown in the CFU, the fluorescence and the XTT assay. Furthermore, an influence mainly on the bottom side of the biofilms could be shown with the aid of fluorescence microscopy and CLSM. Diffusion processes, water channels and plasma flow dynamics might be crucial for this phenomenon. Based on the highly reproducible and new findings, which are generated by the selection of the plasma source and its settings to a certain problem, it would be a decisive step for the industrial use of MIPs in several value chains. For instance, the removal of biofilms from an overgrown surface is of great interest in the food industry. If the inactivation of the bottom side of the biofilms shown in this work also led to its surface detachment, it could be a crucial advantage for the application in the industry.

## Experimental procedures

### Fungal strain and growth conditions

Because of its primarily vertical growth, *C. albicans* SC5314, which is a commonly used strain in laboratory experiments, was grown on Sabouraud agar 4% glucose (Roth, Karlsruhe, Germany) for 24 h at 37°C. Grown colonies were suspended in 10 ml phosphate‐buffered saline (PBS; pH 7.2, according to Sörensen) to an OD_600_ of 0.375–0.385. Afterwards, 1 ml of the suspension was pipetted in 9 ml RPMI medium without bicarbonate (Merck, Darmstadt, Germany). From this inoculum, 200 µl was pipetted into each well of a 96‐well plate and subsequently incubated at 37°C and 80 rpm on a rotary shaker for 90 min to achieve homogeneous oxygen distribution in the biofilm resulting in improved biofilm growth compared to a static grown biofilm. Subsequently, the medium was removed and each well was washed with 200 µl PBS and refilled with 200 µl RPMI medium to remove non‐adhered cells. The plate was incubated at 37°C and 80 rpm on a rotary shaker for 24 h. 96‐well coated polystyrol plates (Sarstedt, Nümbrecht, Germany) were used for biofilm cultivation. For scanning probe measurements (SPM), 12‐well plates with 12 mm coverslips (Sarstedt, Nümbrecht, German) were used. This protocol was kindly provided by the research group of Christiane Yumi Koga‐Ito, Institute of Science and Technology – UNESP, Department of Oral Sciences and Diagnosis (Borges, Lima *et al.*, 2018).

### Plasma source

The MiniMIP worked at a frequency of 2.45 GHz at atmospheric pressure with a forward power in the range of 20–200 W. The microwave discharge was induced in a ceramic tube, which has an inner radius of 0.75 mm and an outer radius of 1.5 mm. The length of the ceramic tube is approximately 31 mm (Fig. 6). The ceramic tube leads completely into the inside of the housing, where it is encased by an aluminium tube. The ignition of the plasma takes place at the front edge of the aluminium tube, and plasma propagation is driven by the surrounding microwave field and the gas flow along the longitudinal axis of the ceramic tube leading to a small plasma plume of about 10 mm in length and 3 mm in diameter outside the device (Baeva, Bösel *et al.*, 2012). In this work, the MiniMIP was operated with a forward power of 40 W and a reverse power of 20 W at a gas flow of 5 slm pure argon gas.

### Plasma treatment of *C. albicans* SC5314 biofilms

After the incubation, mature biofilms (2.1) were washed with PBS (pH 7.2 according to Sörensen) followed by a complete removal of the liquid. Throughout the treatment, the plasma source was attached to a xyz table, holding a constant distance between the source and the biofilm surface. For the treatment, the MiniMIP was horizontally guided into the centre of the biofilm during the treatment and then brought into the desired distance to the biofilm via the computer control software. During the horizontal movement between the treatments of individual well, the plasma source was vertically positioned at the distance of approximately 30 cm (10 times of the treatment distance). A distance of 3 cm was chosen from the beginning of the outer part of the ceramic tube to the surface of the biofilms during the treatment. Each biofilm underwent one of six different treatment times. To avoid dehydration effects, all sample groups were treated in a row. For the colony‐forming units (CFU), the fluorescence LIVE/DEAD assays and the XTT assays, 200 µl PBS (pH 7.2 according to Sörensen) per well was added. Subsequently, the biofilms were mechanically removed from the overgrown surface by repeated pipetting of 200 µl PBS and the resulting cell suspensions were collected. To ensure the transfer of the entire biofilm, this step was repeated three times in total which resulted in a final suspension volume of 600 µl.

### Analytical determinations

First, the influence of the temperature on a biofilm was determined. For temperature measurements, thermal images of the biofilms were acquired directly after plasma treatment with a FLIR thermal imaging camera (FLIR Systems, Frankfurt am Main, Germany) at distance of 20 cm. The temperature had no significant influence on the biofilms (data not shown). The viability of a *C. albicans* biofilm after a plasma treatment was analysed by counting the viable number of microorganisms via the CFU method. Therefore, a 1:10 serial dilution of the samples with maximum recovery diluent (MRD, 0.85% NaCl, 1% tryptone) was performed. Controls and the samples were diluted 1:10 000 and 1:1000 respectively. Each dilution step was plated on Sabouraud agar by pipetting 10 µl per dilution onto the plate and spread out using the tilting technique. Subsequently, the plates were incubated at 37°C for 24 h. The colonies of the dilution levels were counted, and the CFU/ml was calculated as follows:

(1) $$\text{CFU/ml}=\frac{10^{x}}{v}\times \frac{\sum c_{y}+\sum c_{y+1}}{n_{y}+0,1n_{y+1}}$$

10^x^ = the dilution factor for the lowest dilution; v = the volume of diluted cell suspension per plate in ml; ∑c_y_ = the total number of colonies on all (n_y_) plates of the lowest evaluated dilution level 10^–^ *^x^*; ∑c_y+1_ = the total number of colonies on all (n_y_ + 1) plates of the next highest dilution level evaluated 10^–(^ *^x^* ^+1)^ (Bast, 2001).

After the calculation (1), the reduction factor (RF) was determined as follows:

(2) $$\text{RF}={\text{MV}}_{\text{k}}\log_{10}-{\text{MV}}_{\text{p}}\log_{10}$$

MV_k_log_10_ = the mean value of the CFU/ml of the reference group. MV_p_log10 = the mean value of the CFU/ml of the treated specimens.

For the final illustration of the data points, the weighted mean value of the total population was used with the formula:

$$\tilde{x}=\frac{\sum_{i=1}^{n}p_{i}x_{i}}{\sum_{i}p_{i}}=\frac{\left[p\right.\left.x\right]}{\left[p\right]}$$

The weight $p_{i}$ is calculated according to the following formula:

$$p_{i}=k\times \frac{1}{\sigma_{i}^{2}}$$

where *k* = arbitrary constant. This ensures that the weight of the mean values is not included in the calculation as values. The weighted mean value has the advantage compared to the arithmetic mean value that it is more resistant to aberrations (Gränicher, 1994).

The propagation of error was calculated for each treatment group. This finally resulted in four different error propagations for each treatment time from which the weighted error was calculated and used as error bars in the illustration (Gränicher, 1994). The experiments were repeated fourfold with n = 6.

### Fluorescence LIVE/DEAD assay

The LIVE/DEAD BacLight™ Bacterial Viability Kit (Thermo Scientific, Waltham, USA) was prepared according to product instructions. Subsequently, 0.9 µl of the mixture was added to 300 µl of the sample solution (2.3) followed by an incubation on a rotary shaker in the dark at room temperature for 20 min. A fluorescence microplate reader (Varioskan Flash®, Thermo Scientific, Waltham, USA) was used to determine the fluorescence of each well of a 96‐well plate with an excitation wavelength of 470 nm and an emission wavelength of 530 nm (G, green) or 630 nm (R, red). Conclusively, a ratio G/R was calculated by dividing the fluorescence intensity value of green fluorescence by the value of red fluorescence.

### XTT assay

A colorimetric assay was used to determine the cell viability after plasma treatment (XTT Cell Proliferation Assay Kit; AppliChem, St. Louis, MO, USA). Therefore, XTT was applied to reveal the cell viability as a function of redox potential, which arises from a trans‐plasma membrane electron transport (Scudiero, Shoemaker *et al.*, 1988). The sterile activation solution, which contains N‐methyl dibenzopyrazine methyl sulphate (PMS) as an intermediate electron carrier, and the XTT solution were mixed 1:50. For each well, these mixtures were added at a ratio of 1:3 to the sample solution (2.3). The 96‐well plate was incubated at 37°C with continuous horizontal shaking (80 rpm) in the dark for 2 h. After the incubation time, 96‐well plates were scanned at a wavelength of 470 nm using the Varioskan Flash^®^ device. The obtained values were blank‐corrected using XTT and activation solution mix without sample. The experiments were repeated fourfold with n = 6.

### Fluorescence microscopy

Black 96‐well plates with a glass bottom (PerkinElmer, Hamburg, Germany) were used for fluorescence microscopy. To avoid dehydration of the biofilms, plasma‐treated biofilms were resuspended in 300 µl of 0.85% NaCl after treatment. The LIVE/DEAD BacLight™ Bacterial Viability Kit was used as previously described (2.5). Epifluorescence images were acquired using Operetta CLS High‐Content Imager (PerkinElmer, Hamburg, Germany) using a 5× objective (air, NA = 0.16, Zeiss, Oberkochen, Germany). Depending on the experiment, several fields of view were recorded and combined in the software. SYTO™ 9 was excited by a 475 nm (110 mW) LED, and the fluorescence was collected with a 525 ± 25 nm band‐pass filter. Propidium iodide was excited by a 550 nm (170 mW) LED, and the emission light was collected with a 610 ± 40 nm band‐pass filter. A laser autofocus (785 nm) was available for all measurements. The images were displayed using Harmony 4.6 software.

### Confocal laser scanning microscopy (CLSM)

Biofilms were cultivated (2.1), plasma‐treated (2.3) and LIVE/DEAD (2.5)‐stained as previously described. The supernatant was removed after the staining and the washing procedure. Subsequently, the biofilms were analysed using a Zeiss LSM 510 microscope (Carl Zeiss, Jena, Germany) equipped with a 10× objective (air, NA = 0.1). The filter and detector settings were adapted to the fluorescent dyes SYTO™ 9 and propidium iodide. The dyes were excited by an argon laser at 488 nm and the emission was collected at 505–530 nm (band‐pass filter) and 650 nm (long‐pass filter) respectively. Three‐dimensional images were acquired using the ZEN 2009 software (Carl Zeiss) with an area of 1272.2 µm × 1272.2 µm and z‐stack sections of 5.5 µm.

### Atomic force microscopy

For topographic atomic force microscopy (AFM), coverslips were placed on a gel‐like mass Gelrite™ (Duchefa, Haarlem, Netherlands), which avoids unwanted adherence on the bottom side of the coverslip. Because of its rapid curing process, 50 ml Gelrite was autoclaved and directly used hereafter. For the preparation of the liquid mass, a 12‐well plate was used, which was filled with 200 µl per well. While it cooled down, coverslips were placed at the surface of the hardening mass. The biofilms were cultivated as described above (2.1), except that 1 ml of the *C. albicans*–RPMI mix was pipetted to each well until the coverslips were completely topped with the mix. Subsequently, the 12‐well plates were incubated at 37°C and 80 rpm on a rotary shaker for 90 min. Hereafter, the biofilms underwent additional washing steps with 0.85% NaCl. The biofilms were stored overnight in 37°C to ensure the growth of enough biofilm mass. The cultivation medium was removed, and the biofilm‐overgrown coverslips were repeatedly washed with 1 ml 0.85% NaCl the following day.

For the experiments, samples were treated 30 s and 60 s with the MiniMIP. Dehydration of the coverslips before AFM analysis was avoided by using a humidity chamber. The AFM measurements were carried out on a DI CP II SPM (Veeco, Plainview, USA), which was mounted on a vibration‐free object table (TS‐150, Table Stable, Zwillikon, Switzerland). The set‐up was mounted on an optical bench encased by an additional acoustic protection. The AFM was equipped with a linearized piezo scanner, on which the coverslips were mounted with a metal sample holder with leading tabs. The samples were measured using cantilevers with nominal spring constant of *k* = 0.1–0.6 N × m^2^ in contact mode, a frequency of 0.4 Hz and set point = 8 N/m^2^ with a picture size of 20 µm^2^. Pictures were edited with Gwyddion (Czech Metrology Institute, Brno, Czech Republic).

### Optical emission spectroscopy (OES)

For stable OES measurements, the plasma device was left in operation for 30 min until condensation water escaped from the gas pipes and the effluent was optimally adjusted. An optical fibre with an internal diameter of 400 µm was connected to the USB compact spectrometer AvaSpec 2048 (Apeldoorn, Netherlands), which was connected to a laptop. For better focusing, the optical fibre was clamped within a holding bracket. For measurement correction, a black cap was placed on the tip of the fibre optic cable and the dark current was determined. Afterwards, the cap was removed, the fibre optic cable was placed in front of the effluent, and the spectra were measured. The measurement was done for the MiniMIP and the kINPen09, an already commercially used radiofrequency plasma source, for comparison of the OES. The data were read out using matlab ^®^ (MathWorks^®^, Natick, MA, USA) and evaluated and displayed visually as diagrams using Excel.

## Conflict of interest

None declared.

## References

[mbt213459-bib-0001] Al‐Fattani, M.A. , and Douglas, L.J. (2006) Biofilm matrix of *Candida albicans* and *Candida tropicalis*: chemical composition and role in drug resistance. J Med Microbiol 55: 999–1008. 16849719 10.1099/jmm.0.46569-0

[mbt213459-bib-0002] Alkawareek, M.Y. , Algwari, Q.T. , Laverty, G. , Gorman, S.P. , Graham, W.G. , O'Connell, D. , and Gilmore, B.F. (2012) Eradication of Pseudomonas aeruginosa biofilms by atmospheric pressure non‐thermal plasma. PLoS ONE 7: e44289. 22952948 10.1371/journal.pone.0044289 PMC3432087

[mbt213459-bib-0003] Anderson, J.B. (2005) Evolution of antifungal‐drug resistance: mechanisms and pathogen fitness. Nat Rev Microbiol 3: 547–556. 15953931 10.1038/nrmicro1179

[mbt213459-bib-0004] Baeva, M. , Bösel, A. , Ehlbeck, J. , and Loffhagen, D. (2012) Modeling of microwave‐induced plasma in argon at atmospheric pressure. Phys Rev E 85: 056404. 10.1103/PhysRevE.85.056404 23004876

[mbt213459-bib-0005] Baillie, G.S. , and Douglas, L.J. (2000) Matrix polymers of Candida biofilms and their possible role in biofilm resistance to antifungal agents. J Antimicrob Chemother 46: 397–403. 10980166 10.1093/jac/46.3.397

[mbt213459-bib-0006] Banaschik, R. , Lukeš, P. , Jablonowski, H. , Hammer, M.U. , Weltmann, K.D. , and Kolb, J.F. (2015) Potential of pulsed corona discharges generated in water for the degradation of persistent pharmaceutical residues. Water Res 84: 127–135. 26218466 10.1016/j.watres.2015.07.018

[mbt213459-bib-0007] Baroch, P. , and Saito, N. (2011) Dielectric barrier discharge system with catalytically active porous segment for improvement of water treatment.

[mbt213459-bib-0008] Bast, E. (2001) Mikrobiologische methoden, 2nd edn Munich, Germany: Elsevier, p. 429.

[mbt213459-bib-0009] Battey, A.S. , Duffy, S. , and Schaffner, D.W. (2002) Modeling yeast spoilage in cold‐filled ready‐to‐drink beverages with *Saccharomyces cerevisiae*, *Zygosaccharomyces bailii*, and *Candida lipolytica* . Appl Environ Microbiol 68: 1901–1906. 11916710 10.1128/AEM.68.4.1901-1906.2002 PMC123824

[mbt213459-bib-0010] Bekeschus, S. , Schmidt, A. , Weltmann, K.D. , and Woedtke, T. (2016) The plasma jet kINPen – a powerful tool for wound healing. Clinical Plasma Medicine 4: 19–28.

[mbt213459-bib-0011] Bolgova, E.S. , Saprykina, M.N. , and Goncharuk, V.V. (2017) Optimal recultivation conditions of *Candida albicans* staying in non‐culturable state. J Water Chem Technol 39: 305–309.

[mbt213459-bib-0012] Bora, B. , Aguilera, A. , Jain, J. , Avaria, G. , Moreno, J. , Gupta, S.B. , and Soto, L. (2018) Development, characterizations, and applications of a hand touchable DC plasma needle for biomedical investigation. IEEE Trans Plasma Sci 46: 1768–1774.

[mbt213459-bib-0013] Borges, A.C. , Lima, G.D.G. , Nishime, T.M.C. , Gontijo, A.V.L. , Kostov, K.G. , and Koga‐Ito, C.Y. (2018) Amplitude‐modulated cold atmospheric pressure plasma jet for treatment of oral candidiasis: in vivo study. PLoS ONE 13: e0199832. 29949638 10.1371/journal.pone.0199832 PMC6021106

[mbt213459-bib-0014] Bridier, A. , Dubois‐Brissonnet, F. , Greub, G. , Thomas, V. , and Briandet, R. (2011) Dynamics of the action of biocides in *Pseudomonas aeruginosa* biofilms. Antimicrob Agents Chemother 55: 2648–2654. 21422224 10.1128/AAC.01760-10 PMC3101418

[mbt213459-bib-0015] Broekaert, J.A.C. , and Engel, U. (2006) Microwave‐induced plasma systems in atomic spectroscopy. Encyclopedia of Analytical Chemistry 1: 1–81.

[mbt213459-bib-0016] Brugnoni, L.I. , Lozano, J.E. , and Cubitto, M.A. (2007) Potential of yeast isolated from apple juice to adhere to stainless steel surfaces in the apple juice processing industry. Food Res Int 40: 332–340.

[mbt213459-bib-0017] Centers for Disease Control and Prevention (2016) National Outbreak Reporting System (NORS).

[mbt213459-bib-0018] Cowen, L.E. (2008) The evolution of fungal drug resistance: modulating the trajectory from genotype to phenotype. Nat Rev Microbiol 6: 187–198. 18246082 10.1038/nrmicro1835

[mbt213459-bib-0019] Criado, M.T. , Suarez, B. , and Ferreiros, C.M. (1994) The importance of bacterial adhesion in the dairy‐industry. Food Technology 48: 123–126.

[mbt213459-bib-0020] Delben, J.A. , Zago, C.E. , Tyhovych, N. , Duarte, S. , and Vergani, C.E. (2016) Effect of atmospheric‐pressure cold plasma on pathogenic oral biofilms and in vitro reconstituted oral epithelium. PLoS ONE 11: e0155427. 27224027 10.1371/journal.pone.0155427 PMC4880209

[mbt213459-bib-0021] Drost, H. (1980) Plasmachemie. Prozesse der chemischen Stoffwandlung unter Plasma‐Bedingungen. Zeitschrift für Chemie 20: 420.

[mbt213459-bib-0022] Ehlbeck, J. , Brandenburg, R. , von Woedtke, T. , Krohmann, U. , Stieber, M. , and Weltmann, K. D. (2008) PLASMOSE – antimicrobial effects of modular atmospheric plasma sources. GMS Krankenhhyg Interdiszip 3: Doc14. PMC2831527 20204116

[mbt213459-bib-0023] Ehlbeck, J. , Schnabel, U. , Polak, M. , Winter, J. , von Woedtke, T. , Brandenburg, R. , *et al.* (2011) Low temperature atmospheric pressure plasma sources for microbial decontamination. J Phys D Appl Phys 44: 013002.

[mbt213459-bib-0024] Ermolaeva, S.A. , Sysolyatina, E.V. , and Gintsburg, A.L. (2015) Atmospheric pressure nonthermal plasmas for bacterial biofilm prevention and eradication. Biointerphases 10: 029404. 25869456 10.1116/1.4914382

[mbt213459-bib-0025] European Centre for Disease Prevention and Control , and European Food Safety Authority (2017) The European Union summary report on trends and sources of zoonoses, zoonotic agents and food‐borne outbreaks in 2016. EFSA J 15: 228. 10.2903/j.efsa.2017.5077 PMC7009962 32625371

[mbt213459-bib-0026] Flynn, P.B. , Higginbotham, S. , Alshraiedeh, N.H. , Gorman, S.P. , Graham, W.G. , and Gilmore, B.F. (2015) Bactericidal efficacy of atmospheric pressure non‐thermal plasma (APNTP) against the ESKAPE pathogens. Int J Antimicrob Agents 46: 101–107. 25963338 10.1016/j.ijantimicag.2015.02.026

[mbt213459-bib-0027] Fricke, K. , Koban, I. , Tresp, H. , Jablonowski, L. , Schroder, K. , Kramer, A. , *et al.* (2012) Atmospheric pressure plasma: a high‐performance tool for the efficient removal of biofilms. PLoS ONE 7: e42539. 22880025 10.1371/journal.pone.0042539 PMC3412829

[mbt213459-bib-0028] Fridman, G. , Friedman, G. , Gutsol, A. , Shekhter, A.B. , Vasilets, V.N. , and Fridman, A. (2008) Applied plasma medicine. Plasma Processes Polym 5: 503–533.

[mbt213459-bib-0029] Ghaffari, A. , Jalili, R. , Ghaffari, M. , Miller, C. , and Ghahary, A. (2007) Efficacy of gaseous nitric oxide in the treatment of skin and soft tissue infections. Wound Repair Regen 15: 368–377. 17537124 10.1111/j.1524-475X.2007.00239.x

[mbt213459-bib-0030] Gilmore, B.F. , Flynn, P.B. , O'Brien, S. , Hickok, N. , Freeman, T. , and Bourke, P. (2018) Cold plasmas for biofilm control: opportunities and challenges. Trends Biotechnol 36: 627–638. 29729997 10.1016/j.tibtech.2018.03.007 PMC12168448

[mbt213459-bib-0031] Goree, J. , Liu, B. , Drake, D. , and Stoffels, E. (2006) Killing of S‐mutans bacteria using a plasma needle at atmospheric pressure. IEEE Trans Plasma Sci 34: 1317–1324.

[mbt213459-bib-0032] Gränicher, W.H.H. (1994) Messung beendet – was nun? Hochschulverlag AG der ETH Zürich 6‐4‐6‐9.

[mbt213459-bib-0033] Handorf, O. , Weihe, T. , Bekeschus, S. , Graf, A.C. , Schnabel, U. , Riedel, K. , and Ehlbeck, J. (2018) Non‐thermal plasma jet treatment negatively affects viability and structure of *C. albicans* SC5314 biofilms. Appl Environ Microbiol. 84: e01163-18. 10.1128/AEM.01163-18 PMC6193392 30143511

[mbt213459-bib-0034] Herald, P.J. , and Zottola, E.A. (1988) Attachment of listeria‐monocytogenes to stainless‐steel surfaces at various temperatures and pH values. J Food Sci 53: 1549.

[mbt213459-bib-0035] Isbary, G. , Morfill, G. , Schmidt, H.U. , Georgi, M. , Ramrath, K. , Heinlin, J. , *et al.* (2010) A first prospective randomized controlled trial to decrease bacterial load using cold atmospheric argon plasma on chronic wounds in patients. Br J Dermatol 163: 78–82. 20222930 10.1111/j.1365-2133.2010.09744.x

[mbt213459-bib-0036] Jang, H.C. , Rusconi, R. , and Stocker, R. (2017) Biofilm disruption by an air bubble reveals heterogeneous age‐dependent detachment patterns dictated by initial extracellular matrix distribution. NPJ Biofilms Microbiomes 3: 6. 28649407 10.1038/s41522-017-0014-5 PMC5460265

[mbt213459-bib-0037] Jia, H.J. , Kuraseko, H. , and Kondo, M. (2008) A microwave‐induced plasma source: characterization and application for the fast deposition of crystalline silicon films. J Appl Phys 103: 024904.

[mbt213459-bib-0038] Jovicevic, S. , Ivkovic, M. , Pavlovic, Z. , and Konjevic, N. (2000) Parametric study of an atmospheric pressure microwave‐induced plasma of the mini MIP torch – I. Two‐dimensional spatially resolved electron‐number density measurements. Spectrochim Acta B Atomic Spectroscopy 55: 1879–1893.

[mbt213459-bib-0039] Jutkina, J. , Marathe, N.P. , Flach, C.F. , and Larsson, D.G.J. (2018) Antibiotics and common antibacterial biocides stimulate horizontal transfer of resistance at low concentrations. Sci Total Environ 616: 172–178. 29112840 10.1016/j.scitotenv.2017.10.312

[mbt213459-bib-0040] Kabir, M.A. , Hussain, M.A. , and Ahmad, Z. (2012) *Candida albicans*: a model organism for studying fungal pathogens. ISRN Microbiol 2012: 538694. 23762753 10.5402/2012/538694 PMC3671685

[mbt213459-bib-0041] Kerekes, E.B. , Vidács, A. , Jenei, J.T. , Gömöri, C. , Takó, M. , Muthusamy, C. , *et al.* (2015) Essential oils against bacterial biofilm formation and quorum sensing of food‐borne pathogens and spoilage microorganisms. The Battle Against Microbial Pathogens: Basic Science, Technological Advances and Educational Programs 1.

[mbt213459-bib-0042] Khan, M.S.I. , Lee, E.J. , and Kim, Y.J. (2016) A submerged dielectric barrier discharge plasma inactivation mechanism of biofilms produced by *Escherichia coli* O157: H7, *Cronobacter sakazakii*, and *Staphylococcus aureus* . Sci Rep 6: 37072.

[mbt213459-bib-0043] Kieft, I.E. , Darios, D. , Roks, A.J.M. , and Stoffels, E. (2005) Plasma treatment of mammalian vascular cells: a quantitative description. IEEE Trans Plasma Sci 33: 771–775.

[mbt213459-bib-0044] Kieft, I.E. , Kurdi, M. , and Stoffels, E. (2006) Reattachment and apoptosis after plasma‐needle treatment of cultured cells. IEEE Trans Plasma Sci 34: 1331–1336.

[mbt213459-bib-0045] Klampfl, T.G. , Isbary, G. , Shimizu, T. , Li, Y.F. , Zimmermann, J.L. , Stolz, W. , *et al.* (2012) Cold atmospheric air plasma sterilization against spores and other microorganisms of clinical interest. Appl Environ Microbiol 78: 5077–5082. 22582068 10.1128/AEM.00583-12 PMC3416436

[mbt213459-bib-0046] Koban, I. , Matthes, R. , Hubner, N.O. , Welk, A. , Meisel, P. , Holtfreter, B. , *et al.* (2010) Treatment of *Candida albicans* biofilms with low‐temperature plasma induced by dielectric barrier discharge and atmospheric pressure plasma jet. New J Phys 12: 073039.

[mbt213459-bib-0047] Koban, I. , Holtfreter, B. , Hubner, N.O. , Matthes, R. , Sietmann, R. , Kindel, E. , *et al.* (2011) Antimicrobial efficacy of non‐thermal plasma in comparison to chlorhexidine against dental biofilms on titanium discs in vitro – proof of principle experiment. J Clin Periodontol 38: 956–965. 21762196 10.1111/j.1600-051X.2011.01740.x

[mbt213459-bib-0048] Kumar, C.G. , and Anand, S.K. (1998) Significance of microbial biofilms in food industry: a review. Int J Food Microbiol 42: 9–27. 9706794 10.1016/s0168-1605(98)00060-9

[mbt213459-bib-0049] Lademann, J. , Ulrich, C. , Patzelt, A. , Richter, H. , Kluschke, F. , Klebes, M. , *et al.* (2013) Risk assessment of the application of tissue‐tolerable plasma on human skin. Clin Plasma Med 1: 5–10.

[mbt213459-bib-0050] LaFleur, M.D. , Kumamoto, C.A. , and Lewis, K. (2006) *Candida albicans* biofilms produce antifungal‐tolerant persister cells. Antimicrob Agents Chemother 50: 3839–3846. 16923951 10.1128/AAC.00684-06 PMC1635216

[mbt213459-bib-0051] Laroussi, M. , Alexeff, I. , and Kang, W.L. (2000) Biological decontamination by nonthermal plasmas. IEEE Trans Plasma Sci 28: 184–188.

[mbt213459-bib-0052] Laroussi, M. , Mendis, D.A. , and Rosenberg, M. (2003) Plasma interaction with microbes. New J Phys 5: 41.

[mbt213459-bib-0053] Liguori, A. , Cochis, A. , Stancampiano, A. , Laurita, R. , Azzimonti, B. , Sorrentino, R. , *et al.* (2017) Cold atmospheric plasma treatment affects early bacterial adhesion and decontamination of soft reline palatal obturators. Clinical Plasma Medicine 7–8: 36–45.

[mbt213459-bib-0054] Liu, Y. , and Tay, J.H. (2002) The essential role of hydrodynamic shear force in the formation of biofilm and granular sludge. Water Res 36: 1653–1665. 12044065 10.1016/s0043-1354(01)00379-7

[mbt213459-bib-0055] Lorenzini, M. , Simonato, B. , Slaghenaufi, D. , Ugliano, M. , and Zapparoli, G. (2019) Assessment of yeasts for apple juice fermentation and production of cider volatile compounds. Lwt Food Sci Technol 99: 224–230.

[mbt213459-bib-0056] Loureiro, V. , and Malfeito‐Ferreira, M. (2003) Spoilage yeasts in the wine industry. Int J Food Microbiol 86: 23–50. 12892920 10.1016/s0168-1605(03)00246-0

[mbt213459-bib-0057] Machala, Z. , Chladekova, L. , and Pelach, M. (2010) Plasma agents in bio‐decontamination by dc discharges in atmospheric air. J Phys D Appl Phys 43: 222001.

[mbt213459-bib-0058] Mafu, A.A. , Roy, D. , Goulet, J. , and Magny, P. (1990) Attachment of listeria‐monocytogenes to stainless‐steel, glass, polypropylene, and rubber surfaces after short contact times. J Food Prot 53: 742–746. 31018308 10.4315/0362-028X-53.9.742

[mbt213459-bib-0059] Maisch, T. , Shimizu, T. , Isbary, G. , Heinlin, J. , Karrer, S. , Klampfl, T.G. , et al (2012) Contact‐free inactivation of *Candida albicans* biofilms by cold atmospheric air plasma. Appl Environ Microbiol 78: 4242–4247. 22467505 10.1128/AEM.07235-11 PMC3370520

[mbt213459-bib-0060] Mann, M.S. , Schnabel, U. , Weihe, T. , Weltmann, K.D. , and Woedtke, T. (2015) A reference technique to compare the antimicrobial properties of atmospheric pressure plasma sources. Plasma Med 5: 27–47.

[mbt213459-bib-0061] Meireles, A. , Borges, A. , Giaouris, E. , and Simoes, M. (2016) The current knowledge on the application of anti‐biofilm enzymes in the food industry. Food Res Int 86: 140–146.

[mbt213459-bib-0062] Misra, N.N. , Tiwari, B.K. , Raghavarao, K.S.M.S. , and Cullen, P.J. (2011) Nonthermal plasma inactivation of food‐borne pathogens. Food Eng Rev 3: 159–170.

[mbt213459-bib-0063] Modic, M. , McLeod, N.P. , Sutton, J.M. , and Walsh, J.L. (2017) Cold atmospheric pressure plasma elimination of clinically important single‐ and mixed‐species biofilms. Int J Antimicrob Agents 49: 375–378. 28161488 10.1016/j.ijantimicag.2016.11.022

[mbt213459-bib-0064] Mohammed, R.K. , and Abas, H.N. (2018) Bactericidal effect of needle plasma system on *Pseudomonas aeruginosa* . Iran J Sci Technol 42: 1725–1733.

[mbt213459-bib-0065] Morata, A. , and Loira, I. (2017) Yeast‐ Industrial Applications. London, UK: Intech Open.

[mbt213459-bib-0066] Müller, S. , Zahn, R.J. , Koburger, T. , and Weltmann, K.D. (2010) Smell reduction and disinfection of textile materials by dielectric barrier discharges. Natural Science 2: 1044–1048.

[mbt213459-bib-0067] Niemira, B.A. (2012) Cold plasma decontamination of foods. Ann Rev Food Sci Technol 3: 125–142. 22149075 10.1146/annurev-food-022811-101132

[mbt213459-bib-0068] Nobile, C.J. , and Johnson, A.D. (2015) *Candida albicans* biofilms and human disease. Annu Rev Microbiol 69(69): 71–92. 26488273 10.1146/annurev-micro-091014-104330 PMC4930275

[mbt213459-bib-0069] Notermanns, S. , Dormans, J.A.M.A. , and Mead, G.C. (1991) Contribution of surface attachment to the establishment of micro‐organisms in food processing plants: a review. J Bioadhesion Biofilm Res 5: 21–36.

[mbt213459-bib-0070] Oehmigen, K. , Hähnel, M. , Brandenburg, R. , Wilke, C. , Weltmann, K.D. , and von Woedtke, T. (2010) The role of acidification for antimicrobial activity of atmospheric pressure plasma in liquids. Plasma Processes Polym 7: 250–257.

[mbt213459-bib-0071] Offerhaus, B. , Lackmann, J.W. , Kogelheide, F. , Bracht, V. , Smith, R. , Bibinov, N. , et al (2017) Spatially resolved measurements of the physical plasma parameters and the chemical modifications in a twin surface dielectric barrier discharge for gas flow purification. Plasma Processes Polym 14: 1600255.

[mbt213459-bib-0072] Pei, X. , Lu, X. , Liu, J. , Liu, D. , Yang, Y. , Ostrikov, K. , et al (2012) Inactivation of a 25.5 mu m *Enterococcus faecalis* biofilm by a room‐temperature, battery‐operated, handheld air plasma jet. J Phys D Appl Phys 45: 165205.

[mbt213459-bib-0073] Pietsch, G.J. (2001) Peculiarities of dielectric barrier discharges. Contrib Plasma Phys 41: 620–628.

[mbt213459-bib-0074] Pipa, A.V. , Andrasch, M. , Rackow, K. , Ehlbeck, J. , and Weltmann, K.D. (2012) Observation of microwave volume plasma ignition in ambient air. Plasma Sources Sci Technol 21: 035009.

[mbt213459-bib-0075] Ramamurthy, T. , Ghosh, A. , Pazhani, G.P. , and Shinoda, S. (2014) Current perspectives on viable but non‐culturable (VBNC) pathogenic bacteria. Front Public Health 2: 103. 25133139 10.3389/fpubh.2014.00103 PMC4116801

[mbt213459-bib-0076] Saprykina, M.N. , Bolgova, E.S. , and Goncharuk, V.V. (2016) Formation of viable noncultural state of *Candida albicans* . J Water Chem Technol 38: 181–185.

[mbt213459-bib-0077] Sarangapani, C. , Patange, A. , Bourke, P. , Keener, K. , and Cullen, P.J. (2018) Recent advances in the application of cold plasma technology in foods. Ann Rev Food Sci Technol 9: 609–629. 29328805 10.1146/annurev-food-030117-012517

[mbt213459-bib-0078] Schnabel, U. , Andrasch, M. , Weltmann, K.D. , and Ehlbeck, J. (2014) Inactivation of vegetative microorganisms and *Bacillus atrophaeus* endospores by reactive nitrogen species (RNS). Plasma Processes Polym 11: 110–116.

[mbt213459-bib-0079] Schnabel, U. , Handorf, O. , Yarova, K. , Zessin, B. , Zechlin, S. , Sydow, D. , et al (2019) Plasma‐treated air and water‐assessment of synergistic antimicrobial effects for sanitation of food processing surfaces and environment. Foods 8: 55. 10.3390/foods8020055 PMC6406376 30717375

[mbt213459-bib-0080] Scholtz, V. , Pazlarova, J. , Souskova, H. , Khun, J. , and Julak, J. (2015) Nonthermal plasma – a tool for decontamination and disinfection. Biotechnol Adv 33: 1108–1119. 25595663 10.1016/j.biotechadv.2015.01.002

[mbt213459-bib-0081] Scudiero, D.A. , Shoemaker, R.H. , Paull, K.D. , Monks, A. , Tierney, S. , Nofziger, T.H. , et al (1988) Evaluation of a soluble tetrazolium formazan assay for cell‐growth and drug sensitivity in culture using human and other tumor‐cell lines. Can Res 48: 4827–4833. 3409223

[mbt213459-bib-0082] Serra, E. , Hidalgo‐Bastida, L.A. , Verran, J. , Williams, D. , and Malic, S. (2017) Antifungal activity of commercial essential oils and biocides against *Candida albicans* . Pathogens 7: 15. 10.3390/pathogens7010015 PMC5874741 29370147

[mbt213459-bib-0083] Shekhter, A.B. , Serezhenkov, V.A. , Rudenko, T.G. , Pekshev, A.V. , and Vanin, A.F. (2005) Beneficial effect of gaseous nitric oxide on the healing of skin wounds. Nitric Oxide Biol Chem 12: 210–219. 10.1016/j.niox.2005.03.004 15917214

[mbt213459-bib-0084] Simor, M. , Creyghton, Y. , Wypkema, A. , and Zemek, J. (2010) The influence of surface DBD plasma treatment on the adhesion of coatings to high‐tech textiles. J Adhes Sci Technol 24: 77–97.

[mbt213459-bib-0085] Sladek, R.E.J. , Stoffels, E. , Walraven, R. , Tielbeek, P.J.A. , and Koolhoven, R.A. (2004) Plasma treatment of dental cavities: a feasibility study. IEEE Trans Plasma Sci 32: 1540–1543.

[mbt213459-bib-0086] Sladek, R.E.J. , Filoche, S.K. , Sissons, C.H. , and Stoffels, E. (2007) Treatment of *Streptococcus mutans* biofilms with a nonthermal atmospheric plasma. Lett Appl Microbiol 45: 318–323. 17718846 10.1111/j.1472-765X.2007.02194.x

[mbt213459-bib-0087] de Souza, I.A. , Neto, A.B.D. , de Queiroz, J.C.A. , Matamoros, E.P. , Costa, T.H.D. , Feitor, M.C. , *et al.* (2016) Study of the influence of variation in distances between electrodes in spectral DBD plasma excitation. Mater Res Ibero‐Am J Mater 19: 202–206.

[mbt213459-bib-0088] Steenackers, H.P. , Parijs, I. , Dubey, A. , Foster, K.R. , and Vanderleyden, J. (2016) Experimental evolution in biofilm populations. FEMS Microbiol Rev 40: 980–980. 28201751 10.1093/femsre/fuw030 PMC6795384

[mbt213459-bib-0089] Stoffels, E. , Flikweert, A.J. , Stoffels, W.W. , and Kroesen, G.M.W. (2002) Plasma needle: a non‐destructive atmospheric plasma source for fine surface treatment of (bio)materials. Plasma Sources Sci Technol 11: 383–388.

[mbt213459-bib-0090] Sun, Y. , Yu, S. , Sun, P. , Wu, H.Y. , Zhu, W.D. , Liu, W. , et al (2012) Inactivation of candida biofilms by non‐thermal plasma and its enhancement for fungistatic effect of antifungal drugs. PLoS ONE 7: e40629. 22808213 10.1371/journal.pone.0040629 PMC3393702

[mbt213459-bib-0091] Surowsky, B. , Schlüter, O. , and Knorr, D. (2015) Interactions of non‐thermal atmospheric pressure plasma with solid and liquid food systems: a review. Food Eng Rev 7: 82–108.

[mbt213459-bib-0092] Thirumdas, R. , Kothakota, A. , Annapure, U. , Siliveru, K. , Blundell, R. , Gatt, R. , and Valdramidis, V.P. (2018) Plasma activated water (PAW): chemistry, physico‐chemical properties, applications in food and agriculture. Trends Food Sci Technol 77: 21–31.

[mbt213459-bib-0093] Tippayawong, N. , Chaiya, E. , Thanompongchart, P. , and Khongkrapan, P. (2015) Sustainable energy from biogas reforming in a microwave discharge reactor. Proc Eng 118: 120–127.

[mbt213459-bib-0094] Traba, C. , and Liang, J.F. (2015) The inactivation of Staphylococcus aureus biofilms using low‐power argon plasma in a layer‐by‐layer approach. Biofouling 31: 39–48. 25569189 10.1080/08927014.2014.995643 PMC4295521

[mbt213459-bib-0095] Walker, G.M. , and Stewart, G.G. (2016) *Saccharomyces cerevisiae* in the production of fermented beverages. Beverages 2: 30.

[mbt213459-bib-0096] Xu, L. , Tu, Y. , Yu, Y. , Tan, M. , Li, J. , and Chen, H. (2011) Augmented survival of Neisseria gonorrhoeae within biofilms: exposure to atmospheric pressure non‐thermal plasmas. Eur J Clin Microbiol Infect Dis 30: 25–31. 20839022 10.1007/s10096-010-1047-3

[mbt213459-bib-0097] Xu, Z.M. , Shen, J. , Zhang, Z.L. , Ma, J. , Ma, R.H. , Zhao, Y. , *et al.* (2015) Inactivation effects of non‐thermal atmospheric‐pressure helium plasma jet on *Staphylococcus aureus* biofilms. Plasma Processes Polym 12: 827–835.

[mbt213459-bib-0098] Xu, Z.M. , Shen, J. , Cheng, C. , Hu, S.H. , Lan, Y. , and Chu, P.K. (2017) In vitro antimicrobial effects and mechanism of atmospheric‐pressure He/O‐2 plasma jet on *Staphylococcus aureus* biofilm. J Phys D Appl Phys 50: 105201.

[mbt213459-bib-0099] Yusupov, M. , Neyts, E.C. , Khalilov, U. , Snoeckx, R. , van Duin, A.C.T. , and Bogaerts, A. (2012) Atomic‐scale simulations of reactive oxygen plasma species interacting with bacterial cell walls. New J Phys 14: 093043.

[mbt213459-bib-0100] Ziuzina, D. , Boehm, D. , Patil, S. , Cullen, P.J. , and Bourke, P. (2015) Cold plasma inactivation of bacterial biofilms and reduction of quorum sensing regulated virulence factors. PLoS ONE 10: e0138209. 26390435 10.1371/journal.pone.0138209 PMC4577073

